# A second tricilinc polymorph of 6,6′-dieth­oxy-2,2′-[propane-1,2-diylbis(nitrilo­methyl­idyne)]diphenol

**DOI:** 10.1107/S1600536809008137

**Published:** 2009-03-11

**Authors:** Hoong-Kun Fun, Reza Kia, Hadi Kargar, Arezoo Jamshidvand

**Affiliations:** a X-ray Crystallography Unit, School of Physics, Universiti Sains Malaysia, 11800 USM, Penang, Malaysia; b Department of Chemistry, School of Science, Payame Noor University (PNU), Ardakan, Yazd, Iran

## Abstract

The title Schiff base compound, C_21_H_26_N_2_O_4_, is a second triclinic polymorph of a previously reported room-temperature structure [Jia (2009 ▶). *Acta Cryst.* E**65**, o646]. Strong intra­molecular O—H⋯N hydrogen bonds generate *S*(6) ring motifs. Inter­molecular C—H⋯O inter­actions link neighbouring mol­ecules into dimers with an *R*_2_^2^(16) ring motif. The mean planes of the two benzene rings are almost perpendicular to each other, making a dihedral angle of 88.24 (5)°. An inter­esting feature of the crystal structure is the intermolecular short C⋯O [3.1878 (13) Å] contact which is shorter than the sum of the van der Waals radii of the relevant atoms. The crystal structure is further stabilized by inter­molecular C—H⋯π and π–π inter­actions [centroid–centroid distance = 3.7414 (6) Å]. The structure has a stereogenic centre but the space group is centrosymmetric, so the mol­ecule exists as a racemate.

## Related literature

For hydrogen-bond motifs, see: Bernstein *et al.* (1995 ▶). For information on Schiff base ligands, complexes and their applications, see: Calligaris & Randaccio (1987 ▶). For the other polymorph, see: Jia, (2009 ▶). For related structures, see: Li *et al.* (2005 ▶); Bomfim *et al.* (2005 ▶); Glidewell *et al.* (2005, 2006 ▶); Sun *et al.* (2004 ▶); Fun *et al.* (2008 ▶). For bond-length data, see: Allen *et al.* (1987 ▶). For stability of the temperature controller used for data collection, see: Cosier & Glazer (1986 ▶).
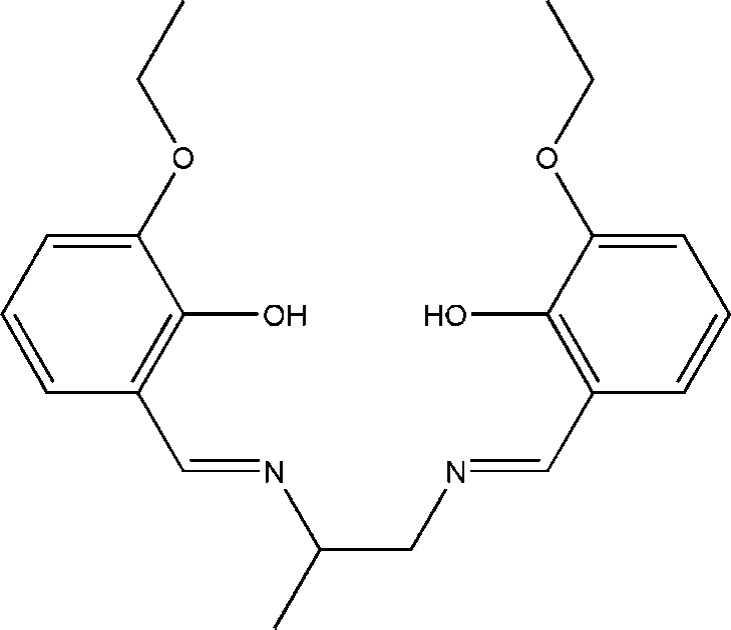


## Experimental

### 

#### Crystal data

C_21_H_26_N_2_O_4_
*M* *_r_* = 370.44
Triclinic, 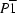

*a* = 8.9729 (2) Å
*b* = 10.7008 (4) Å
*c* = 11.3633 (2) Å
α = 107.432 (1)°
β = 108.487 (1)°
γ = 95.979 (1)°
*V* = 963.03 (5) Å^3^
*Z* = 2
Mo *K*α radiation
μ = 0.09 mm^−1^
*T* = 100 K
0.56 × 0.27 × 0.25 mm

#### Data collection

Bruker SMART APEXII CCD area-detector diffractometer
Absorption correction: multi-scan (**SADABS**; Bruker, 2005 ▶) *T*_min_ = 0.952, *T*_max_ = 0.978
19581 measured reflections
5527 independent reflections
4721 reflections with *I* > 2σ(*I*)
*R*_int_ = 0.026

#### Refinement

*R*[*F* ^2^ > 2σ(*F* ^2^)] = 0.045
*wR*(*F* ^2^) = 0.136
*S* = 1.05
5527 reflections
249 parameters
H-atom parameters constrained
Δρ_max_ = 0.53 e Å^−3^
Δρ_min_ = −0.23 e Å^−3^

### 

Data collection: *APEX2* (Bruker, 2005 ▶); cell refinement: *SAINT* (Bruker, 2005 ▶); data reduction: *SAINT*; program(s) used to solve structure: *SHELXTL* (Sheldrick, 2008 ▶); program(s) used to refine structure: *SHELXTL*; molecular graphics: *SHELXTL*; software used to prepare material for publication: *SHELXTL* and *PLATON* (Spek, 2009 ▶).

## Supplementary Material

Crystal structure: contains datablocks global, I. DOI: 10.1107/S1600536809008137/at2736sup1.cif

Structure factors: contains datablocks I. DOI: 10.1107/S1600536809008137/at2736Isup2.hkl

Additional supplementary materials:  crystallographic information; 3D view; checkCIF report

## Figures and Tables

**Table 1:** Hydrogen-bond geometry (Å, °)

*D*—H⋯*A*	*D*—H	H⋯*A*	*D*⋯*A*	*D*—H⋯*A*
O1—H1⋯N1	0.84	1.83	2.5752 (13)	146
O2—H2⋯N2	0.84	1.88	2.6178 (13)	147
C9—H9*A*⋯O1^i^	0.99	2.49	3.4293 (14)	159
C18—H18b⋯*Cg*1^ii^	0.99	2.98	3.8340 (12)	142
C7—H7*A*⋯*Cg*2^iii^	0.96	2.72	3.5554 (12)	176

Symmetry codes: (i) 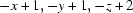; (ii) 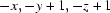; (iii) 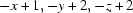. *Cg*1 and *Cg*2 are the centroids of the C1–C6 and C11–C16 benzene rings, respectively.

**Fig. 1.**
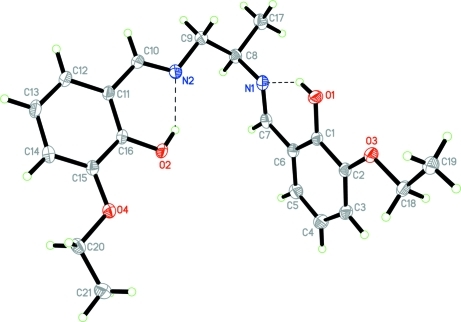
The molecular structure of the title compound with atom labels and 50% probability ellipsoids for non-H atoms. Dashed lines indicate intramolecular O—H···N hydrogen bonds.

**Fig. 2.**
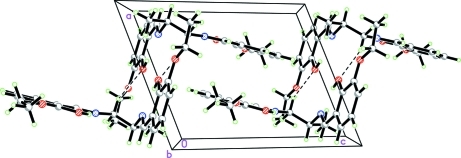
The crystal structure of the title compound, viewed down the b-axis, showing dimer formation by R22(16) ring motif.

## supplementary crystallographic information

### Comment

Schiff bases are one of the most prevalent mixed-donor ligands in the field of coordination chemistry. They play an important role in the development of coordination chemistry related to catalysis and enzymatic reactions, magnetism, and supramolecular architectures (Calligaris & Randaccio, 1987). Structures of Schiff bases derived from substituted benzaldehydes and closely related to the title compound have been reported earlier (Li *et al.*, 2005; Bomfim *et al.*, 2005; Glidewell *et al.*, 2006; Sun *et al.*, 2004; Fun *et al.,* 2008).

The molecule of the title compound (Fig. 1), is a potentially tetradentate Schiff base ligand. The bond lengths (Allen *et al.,* 1987) and angles are comparable to the earlier room-temperature polymorph which was published previously (Jia, 2009). Strong intramolecular O—H···N hydrogen bonds generate *S(6)* ring motifs (Bernstein *et al.*, 1995). Intermolecular C—H···O interactions link neighbouring molecules into dimers with a *R_2_^2^(16)* ring motif (Bernstein *et al.,* 1995). The mean planes of the two benzene rings are almost perpendicular to each other making a dihedral angle of 88.24 (5)°. The interesting feature of the crystal structure is the short C18···O2 [3.1878 (13) Å, symmetry code: 1 - x, 1 - y, 1 - z] contact which is shorter than the sum of the van der Waals radii of the relevant atoms. The crystal structure, is further stabilizd by intermolecular C—H···π and π-π interactions [centroid to centroid distance of 3.7414 (6) Å]. The structure has a stereogenic centre but the space group is centrosymmetric, so the molecule exists as racemate.

### Experimental

The synthetic method has been described earlier (Fun *et al.,* 2008), except that 3-ethoxy salicylaldehyde and 2-methyl-2,3-propanediamine were used as starting materials. Single crystals suitable for *X*-ray diffraction were obtained by evaporation of an ethanol solution at room temperature.

### Refinement

H atoms of the hydroxy groups were positioned by a freely rotating O—H bond and constrained with a fixed distance of 0.84 Å. The rest of the hydrogen atoms were positioned geometrically and refined using a riding model with C—H = 0.95–1.00 Å and U_iso_(H) = 1.2 or 1.5 U_eq_(C). A rotating-group model was applied for the methyl groups.

### Crystal data

**Table:**

C_21_H_26_N_2_O_4_	*Z* = 2
*M**_r_* = 370.44	*F*(000) = 396
Triclinic, *P*1	*D*_x_ = 1.277 Mg m^−^^3^
Hall symbol: -P 1	Mo *K*α radiation, λ = 0.71073 Å
*a* = 8.9729 (2) Å	Cell parameters from 9912 reflections
*b* = 10.7008 (4) Å	θ = 2.5–33.9°
*c* = 11.3633 (2) Å	µ = 0.09 mm^−^^1^
α = 107.432 (1)°	*T* = 100 K
β = 108.487 (1)°	Block, yellow
γ = 95.979 (1)°	0.56 × 0.27 × 0.25 mm
*V* = 963.03 (5) Å^3^	

### Data collection

**Table:**

Bruker SMART APEXII CCD area-detector diffractometer	5527 independent reflections
Radiation source: fine-focus sealed tube	4721 reflections with *I* > 2σ(*I*)
graphite	*R*_int_ = 0.026
φ and ω scans	θ_max_ = 30.0°, θ_min_ = 2.0°
Absorption correction: multi-scan (*SADABS*; Bruker, 2005)	*h* = −12→12
*T*_min_ = 0.952, *T*_max_ = 0.978	*k* = −15→15
19581 measured reflections	*l* = −15→15

### Refinement

**Table:**

Refinement on *F*^2^	Primary atom site location: structure-invariant direct methods
Least-squares matrix: full	Secondary atom site location: difference Fourier map
*R*[*F*^2^ > 2σ(*F*^2^)] = 0.045	Hydrogen site location: inferred from neighbouring sites
*wR*(*F*^2^) = 0.136	H-atom parameters constrained
*S* = 1.05	*w* = 1/[σ^2^(*F*_o_^2^) + (0.0795*P*)^2^ + 0.2658*P*] where *P* = (*F*_o_^2^ + 2*F*_c_^2^)/3
5527 reflections	(Δ/σ)_max_ = 0.001
249 parameters	Δρ_max_ = 0.53 e Å^−^^3^
0 restraints	Δρ_min_ = −0.23 e Å^−^^3^

### Special details

**Table:**

Experimental. The crystal was placed in the cold stream of an Oxford Cyrosystems Cobra open-flow nitrogen cryostat (Cosier & Glazer, 1986) operating at 100.0 (1)K.
Geometry. All esds (except the esd in the dihedral angle between two l.s. planes) are estimated using the full covariance matrix. The cell esds are taken into account individually in the estimation of esds in distances, angles and torsion angles; correlations between esds in cell parameters are only used when they are defined by crystal symmetry. An approximate (isotropic) treatment of cell esds is used for estimating esds involving l.s. planes.
Refinement. Refinement of F^2^ against ALL reflections. The weighted R-factor wR and goodness of fit S are based on F^2^, conventional R-factors R are based on F, with F set to zero for negative F^2^. The threshold expression of F^2^ > 2sigma(F^2^) is used only for calculating R-factors(gt) etc. and is not relevant to the choice of reflections for refinement. R-factors based on F^2^ are statistically about twice as large as those based on F, and R- factors based on ALL data will be even larger.

### Fractional atomic coordinates and isotropic or equivalent isotropic displacement parameters (Å^2^)

**Table:**

	*x*	*y*	*z*	*U*_iso_*/*U*_eq_	
O1	0.45973 (9)	0.68396 (7)	1.04387 (8)	0.02067 (16)	
H1	0.3996	0.6074	0.9989	0.031*	
O2	0.23678 (10)	0.61221 (7)	0.56112 (7)	0.02165 (17)	
H2	0.2199	0.5617	0.6018	0.032*	
O3	0.60593 (9)	0.93704 (7)	1.19014 (7)	0.02040 (16)	
O4	0.30071 (10)	0.74269 (7)	0.41348 (7)	0.02157 (17)	
N1	0.20357 (10)	0.51029 (9)	0.87252 (9)	0.01932 (18)	
N2	0.22170 (11)	0.39204 (9)	0.61688 (9)	0.01981 (18)	
C1	0.37342 (12)	0.77863 (10)	1.03131 (9)	0.01695 (19)	
C2	0.45003 (12)	0.91483 (10)	1.10727 (10)	0.01745 (19)	
C3	0.36454 (13)	1.01381 (10)	1.09335 (10)	0.0202 (2)	
H3A	0.4165	1.1055	1.1421	0.024*	
C4	0.20267 (13)	0.98033 (11)	1.00838 (11)	0.0230 (2)	
H4A	0.1456	1.0492	1.0003	0.028*	
C5	0.12610 (12)	0.84751 (11)	0.93638 (11)	0.0218 (2)	
H5A	0.0158	0.8249	0.8801	0.026*	
C6	0.21104 (12)	0.74568 (10)	0.94623 (10)	0.01782 (19)	
C7	0.13033 (12)	0.60568 (10)	0.86605 (10)	0.0193 (2)	
H7A	0.0211	0.5852	0.8079	0.023*	
C8	0.11726 (12)	0.37212 (10)	0.78846 (10)	0.0197 (2)	
H8A	0.0055	0.3712	0.7325	0.024*	
C9	0.20882 (13)	0.31383 (10)	0.70025 (10)	0.0209 (2)	
H9A	0.3182	0.3127	0.7564	0.025*	
H9B	0.1525	0.2200	0.6432	0.025*	
C10	0.25539 (12)	0.33633 (10)	0.51501 (10)	0.0195 (2)	
H10A	0.2624	0.2448	0.4936	0.023*	
C11	0.28343 (12)	0.40753 (10)	0.43059 (10)	0.01798 (19)	
C12	0.32606 (13)	0.34087 (11)	0.32285 (10)	0.0224 (2)	
H12A	0.3319	0.2492	0.3041	0.027*	
C13	0.35938 (13)	0.40746 (11)	0.24449 (10)	0.0236 (2)	
H13A	0.3881	0.3617	0.1721	0.028*	
C14	0.35108 (12)	0.54260 (11)	0.27131 (10)	0.0211 (2)	
H14A	0.3732	0.5880	0.2165	0.025*	
C15	0.31054 (12)	0.61053 (10)	0.37785 (10)	0.01799 (19)	
C16	0.27562 (11)	0.54322 (10)	0.45865 (9)	0.01716 (19)	
C17	0.11095 (14)	0.29050 (11)	0.87711 (11)	0.0251 (2)	
H17A	0.0583	0.3321	0.9376	0.038*	
H17B	0.2207	0.2884	0.9286	0.038*	
H17C	0.0497	0.1987	0.8220	0.038*	
C18	0.68435 (13)	1.07531 (10)	1.27022 (10)	0.0207 (2)	
H18A	0.6284	1.1129	1.3306	0.025*	
H18B	0.6818	1.1295	1.2129	0.025*	
C19	0.85652 (13)	1.07880 (11)	1.34933 (11)	0.0250 (2)	
H19A	0.9117	1.1714	1.4071	0.038*	
H19B	0.9119	1.0446	1.2887	0.038*	
H19C	0.8576	1.0226	1.4036	0.038*	
C20	0.32265 (13)	0.81235 (11)	0.32824 (11)	0.0228 (2)	
H20A	0.2482	0.7626	0.2357	0.027*	
H20B	0.4345	0.8213	0.3305	0.027*	
C21	0.28754 (16)	0.94927 (12)	0.37877 (13)	0.0284 (2)	
H21A	0.3039	1.0011	0.3242	0.043*	
H21B	0.3602	0.9965	0.4710	0.043*	
H21C	0.1757	0.9390	0.3739	0.043*	

### Atomic displacement parameters (Å^2^)

**Table:**

	*U*^11^	*U*^22^	*U*^33^	*U*^12^	*U*^13^	*U*^23^
O1	0.0213 (3)	0.0161 (3)	0.0204 (4)	0.0042 (3)	0.0039 (3)	0.0045 (3)
O2	0.0338 (4)	0.0181 (3)	0.0173 (3)	0.0072 (3)	0.0154 (3)	0.0053 (3)
O3	0.0215 (3)	0.0164 (3)	0.0181 (3)	0.0023 (3)	0.0042 (3)	0.0027 (3)
O4	0.0309 (4)	0.0184 (3)	0.0173 (3)	0.0041 (3)	0.0113 (3)	0.0066 (3)
N1	0.0206 (4)	0.0199 (4)	0.0153 (4)	0.0007 (3)	0.0065 (3)	0.0044 (3)
N2	0.0234 (4)	0.0187 (4)	0.0165 (4)	0.0036 (3)	0.0070 (3)	0.0059 (3)
C1	0.0202 (4)	0.0180 (4)	0.0139 (4)	0.0042 (3)	0.0082 (3)	0.0053 (3)
C2	0.0204 (4)	0.0183 (4)	0.0145 (4)	0.0037 (3)	0.0085 (3)	0.0049 (3)
C3	0.0257 (5)	0.0191 (4)	0.0187 (5)	0.0064 (4)	0.0124 (4)	0.0055 (4)
C4	0.0251 (5)	0.0247 (5)	0.0240 (5)	0.0110 (4)	0.0132 (4)	0.0090 (4)
C5	0.0199 (4)	0.0271 (5)	0.0209 (5)	0.0080 (4)	0.0096 (4)	0.0087 (4)
C6	0.0191 (4)	0.0202 (4)	0.0153 (4)	0.0039 (3)	0.0084 (3)	0.0057 (3)
C7	0.0190 (4)	0.0231 (5)	0.0150 (4)	0.0018 (3)	0.0070 (3)	0.0058 (4)
C8	0.0195 (4)	0.0194 (4)	0.0169 (4)	0.0010 (3)	0.0047 (3)	0.0050 (4)
C9	0.0264 (5)	0.0188 (4)	0.0178 (4)	0.0055 (4)	0.0078 (4)	0.0070 (4)
C10	0.0226 (4)	0.0166 (4)	0.0164 (4)	0.0043 (3)	0.0055 (4)	0.0038 (3)
C11	0.0194 (4)	0.0181 (4)	0.0140 (4)	0.0041 (3)	0.0052 (3)	0.0033 (3)
C12	0.0276 (5)	0.0212 (5)	0.0169 (4)	0.0079 (4)	0.0088 (4)	0.0033 (4)
C13	0.0272 (5)	0.0277 (5)	0.0155 (4)	0.0083 (4)	0.0103 (4)	0.0037 (4)
C14	0.0218 (5)	0.0262 (5)	0.0152 (4)	0.0041 (4)	0.0075 (4)	0.0066 (4)
C15	0.0184 (4)	0.0188 (4)	0.0146 (4)	0.0026 (3)	0.0052 (3)	0.0043 (3)
C16	0.0185 (4)	0.0185 (4)	0.0125 (4)	0.0036 (3)	0.0059 (3)	0.0029 (3)
C17	0.0282 (5)	0.0245 (5)	0.0222 (5)	0.0000 (4)	0.0093 (4)	0.0096 (4)
C18	0.0247 (5)	0.0160 (4)	0.0181 (4)	0.0019 (3)	0.0081 (4)	0.0021 (3)
C19	0.0253 (5)	0.0213 (5)	0.0222 (5)	0.0020 (4)	0.0049 (4)	0.0042 (4)
C20	0.0276 (5)	0.0240 (5)	0.0205 (5)	0.0041 (4)	0.0116 (4)	0.0105 (4)
C21	0.0377 (6)	0.0249 (5)	0.0295 (6)	0.0082 (4)	0.0168 (5)	0.0140 (4)

### Geometric parameters (Å, °)

**Table:**

O1—C1	1.3514 (12)	C9—H9B	0.9900
O1—H1	0.8400	C10—C11	1.4542 (15)
O2—C16	1.3484 (11)	C10—H10A	0.9500
O2—H2	0.8400	C11—C16	1.4046 (13)
O3—C2	1.3643 (12)	C11—C12	1.4087 (14)
O3—C18	1.4432 (12)	C12—C13	1.3765 (16)
O4—C15	1.3701 (12)	C12—H12A	0.9500
O4—C20	1.4338 (13)	C13—C14	1.4008 (15)
N1—C7	1.2780 (14)	C13—H13A	0.9500
N1—C8	1.4644 (13)	C14—C15	1.3905 (14)
N2—C10	1.2777 (13)	C14—H14A	0.9500
N2—C9	1.4614 (14)	C15—C16	1.4112 (14)
C1—C6	1.4062 (13)	C17—H17A	0.9800
C1—C2	1.4153 (13)	C17—H17B	0.9800
C2—C3	1.3885 (14)	C17—H17C	0.9800
C3—C4	1.4018 (15)	C18—C19	1.5110 (15)
C3—H3A	0.9500	C18—H18A	0.9900
C4—C5	1.3802 (15)	C18—H18B	0.9900
C4—H4A	0.9500	C19—H19A	0.9800
C5—C6	1.4046 (14)	C19—H19B	0.9800
C5—H5A	0.9500	C19—H19C	0.9800
C6—C7	1.4617 (14)	C20—C21	1.5107 (16)
C7—H7A	0.9500	C20—H20A	0.9900
C8—C9	1.5242 (15)	C20—H20B	0.9900
C8—C17	1.5277 (15)	C21—H21A	0.9800
C8—H8A	1.0000	C21—H21B	0.9800
C9—H9A	0.9900	C21—H21C	0.9800
			
C1—O1—H1	109.5	C13—C12—C11	120.54 (9)
C16—O2—H2	109.5	C13—C12—H12A	119.7
C2—O3—C18	116.03 (8)	C11—C12—H12A	119.7
C15—O4—C20	116.95 (8)	C12—C13—C14	120.13 (9)
C7—N1—C8	118.97 (9)	C12—C13—H13A	119.9
C10—N2—C9	117.69 (9)	C14—C13—H13A	119.9
O1—C1—C6	122.09 (9)	C15—C14—C13	120.23 (10)
O1—C1—C2	118.39 (8)	C15—C14—H14A	119.9
C6—C1—C2	119.52 (9)	C13—C14—H14A	119.9
O3—C2—C3	125.27 (9)	O4—C15—C14	124.90 (9)
O3—C2—C1	115.48 (9)	O4—C15—C16	114.95 (8)
C3—C2—C1	119.25 (9)	C14—C15—C16	120.15 (9)
C2—C3—C4	120.94 (9)	O2—C16—C11	122.22 (9)
C2—C3—H3A	119.5	O2—C16—C15	118.56 (9)
C4—C3—H3A	119.5	C11—C16—C15	119.22 (9)
C5—C4—C3	120.10 (10)	C8—C17—H17A	109.5
C5—C4—H4A	119.9	C8—C17—H17B	109.5
C3—C4—H4A	119.9	H17A—C17—H17B	109.5
C4—C5—C6	120.06 (10)	C8—C17—H17C	109.5
C4—C5—H5A	120.0	H17A—C17—H17C	109.5
C6—C5—H5A	120.0	H17B—C17—H17C	109.5
C5—C6—C1	120.10 (9)	O3—C18—C19	107.74 (8)
C5—C6—C7	119.59 (9)	O3—C18—H18A	110.2
C1—C6—C7	120.30 (9)	C19—C18—H18A	110.2
N1—C7—C6	121.39 (9)	O3—C18—H18B	110.2
N1—C7—H7A	119.3	C19—C18—H18B	110.2
C6—C7—H7A	119.3	H18A—C18—H18B	108.5
N1—C8—C9	108.28 (8)	C18—C19—H19A	109.5
N1—C8—C17	108.78 (8)	C18—C19—H19B	109.5
C9—C8—C17	109.96 (9)	H19A—C19—H19B	109.5
N1—C8—H8A	109.9	C18—C19—H19C	109.5
C9—C8—H8A	109.9	H19A—C19—H19C	109.5
C17—C8—H8A	109.9	H19B—C19—H19C	109.5
N2—C9—C8	111.50 (8)	O4—C20—C21	106.98 (9)
N2—C9—H9A	109.3	O4—C20—H20A	110.3
C8—C9—H9A	109.3	C21—C20—H20A	110.3
N2—C9—H9B	109.3	O4—C20—H20B	110.3
C8—C9—H9B	109.3	C21—C20—H20B	110.3
H9A—C9—H9B	108.0	H20A—C20—H20B	108.6
N2—C10—C11	122.60 (9)	C20—C21—H21A	109.5
N2—C10—H10A	118.7	C20—C21—H21B	109.5
C11—C10—H10A	118.7	H21A—C21—H21B	109.5
C16—C11—C12	119.72 (10)	C20—C21—H21C	109.5
C16—C11—C10	120.87 (9)	H21A—C21—H21C	109.5
C12—C11—C10	119.35 (9)	H21B—C21—H21C	109.5
			
C18—O3—C2—C3	1.11 (15)	C17—C8—C9—N2	−178.20 (8)
C18—O3—C2—C1	−178.73 (8)	C9—N2—C10—C11	−175.17 (9)
O1—C1—C2—O3	−1.69 (13)	N2—C10—C11—C16	0.46 (15)
C6—C1—C2—O3	177.98 (9)	N2—C10—C11—C12	177.59 (10)
O1—C1—C2—C3	178.45 (9)	C16—C11—C12—C13	−0.30 (15)
C6—C1—C2—C3	−1.88 (15)	C10—C11—C12—C13	−177.47 (9)
O3—C2—C3—C4	−177.92 (10)	C11—C12—C13—C14	−0.02 (16)
C1—C2—C3—C4	1.92 (15)	C12—C13—C14—C15	0.56 (16)
C2—C3—C4—C5	−0.39 (17)	C20—O4—C15—C14	4.75 (14)
C3—C4—C5—C6	−1.17 (16)	C20—O4—C15—C16	−175.21 (8)
C4—C5—C6—C1	1.18 (16)	C13—C14—C15—O4	179.28 (9)
C4—C5—C6—C7	−177.88 (9)	C13—C14—C15—C16	−0.76 (15)
O1—C1—C6—C5	−179.99 (9)	C12—C11—C16—O2	−179.44 (9)
C2—C1—C6—C5	0.35 (15)	C10—C11—C16—O2	−2.33 (15)
O1—C1—C6—C7	−0.94 (15)	C12—C11—C16—C15	0.10 (14)
C2—C1—C6—C7	179.41 (9)	C10—C11—C16—C15	177.22 (9)
C8—N1—C7—C6	−179.15 (9)	O4—C15—C16—O2	−0.05 (13)
C5—C6—C7—N1	−179.86 (10)	C14—C15—C16—O2	179.99 (9)
C1—C6—C7—N1	1.08 (15)	O4—C15—C16—C11	−179.61 (8)
C7—N1—C8—C9	121.02 (10)	C14—C15—C16—C11	0.43 (14)
C7—N1—C8—C17	−119.50 (10)	C2—O3—C18—C19	−177.40 (9)
C10—N2—C9—C8	−161.52 (9)	C15—O4—C20—C21	173.58 (9)
N1—C8—C9—N2	−59.47 (11)		

### Hydrogen-bond geometry (Å, °)

**Table:**

*D*—H···*A*	*D*—H	H···*A*	*D*···*A*	*D*—H···*A*
O1—H1···N1	0.84	1.83	2.5752 (13)	146
O2—H2···N2	0.84	1.88	2.6178 (13)	147
C9—H9A···O1^i^	0.99	2.49	3.4293 (14)	159
C18—H18b···Cg1^ii^	0.9900	2.9800	3.8340 (12)	142.00
C7—H7A···Cg2^iii^	0.96	2.72	3.5554 (12)	176

Symmetry codes: (i) −*x*+1, −*y*+1, −*z*+2; (ii) −*x*, −*y*+1, −*z*+1; (iii) −*x*+1, −*y*+2, −*z*+2.
